# The capacity to maintain ion and water homeostasis underlies interspecific variation in *Drosophila* cold tolerance

**DOI:** 10.1038/srep18607

**Published:** 2015-12-18

**Authors:** Heath A. MacMillan, Jonas L. Andersen, Shireen A. Davies, Johannes Overgaard

**Affiliations:** 1 Zoophysiology, Department of Bioscience, Aarhus University, Aarhus, Denmark; 2 Institute of Molecular, Cell and Systems Biology, College of Medical, Veterinary and Life Sciences, University of Glasgow, Glasgow, United Kingdom

## Abstract

Many insects, including *Drosophila*, succumb to the physiological effects of chilling at temperatures well above those causing freezing. Low temperature causes a loss of extracellular ion and water homeostasis in such insects, and chill injuries accumulate. Using an integrative and comparative approach, we examined the role of ion and water balance in insect chilling susceptibility/ tolerance. The Malpighian tubules (MT), of chill susceptible *Drosophila* species lost [Na^+^] and [K^+^] selectivity at low temperatures, which contributed to a loss of Na^+^ and water balance and a deleterious increase in extracellular [K^+^]. By contrast, the tubules of chill tolerant *Drosophila* species maintained their MT ion selectivity, maintained stable extracellular ion concentrations, and thereby avoided injury. The most tolerant species were able to modulate ion balance while in a cold-induced coma and this ongoing physiological acclimation process allowed some individuals of the tolerant species to recover from chill coma during low temperature exposure. Accordingly, differences in the ability to maintain homeostatic control of water and ion balance at low temperature may explain large parts of the wide intra- and interspecific variation in insect chilling tolerance.

Insects account for more than 75% of all animal species on Earth, are present in virtually every ecosystem, and play important ecological and societal roles as pollinators, pests, disease carriers, and predators of “unwanted” species^1^,^2^. The geographical distributions of individual insect species correlate remarkably well with their ability to tolerate abiotic variability. In particular, thermal and desiccation tolerance are considered good predictors of species’ distribution^3^,^4^,^5^,^6^,^7^,^8^. The association between species distribution and cold tolerance is particularly strong and the importance of environmental tolerance is also inferred from recent changes in geographical distribution in response to the current global warming^9^,^10^,^11^,^12^.

The majority of insect species are unable to tolerate freezing or endure very low subzero temperatures in a supercooled state^13^,^14^,^15^. Instead these “chill susceptible” insects are vulnerable to relatively mild cold exposures and accumulating evidence suggests that chill susceptibility is principally related to an inability to maintain ion and water homeostasis at low temperatures^16^,^17^,^18^. This loss of balance is thought to occur because active transport systems are suppressed at low temperature to a point at which they are unable to sufficiently counter passive leak of ions down their concentration gradients across membranes and epithelia^16^,^17^,^18^,^19^,^20^,^21^,^22^,^23^,^24^,^25^. Briefly, the hemolymph of many insects is high in [Na^+^] and low in [K^+^], meaning Na^+^ ions will tend to leak into the gut lumen or cell cytoplasm while K^+^ ions do the opposite (i.e. into the hemolymph). At benign temperatures, passive ion movements to and from the hemolymph are regulated by the energy-demanding ion secretion of the Malpighian tubule (MT) and simultaneous ion re-absorption of the hindgut^26^,^27^. Lowering temperature causes net Na^+^ leak away from the hemolymph and as water follows Na^+^ osmotically, it causes a simultaneous reduction in hemolymph volume that concentrates [K^+^] in the remaining extracellular fluid. The resulting increase in hemolymph [K^+^] depolarizes cell resting potential (which is highly dependent on the [K^+^] gradient^19^,^28^,^29^,^30^) and this depolarization may be a primary cause of cold-induced injury^16^,^31^,^32^. According to this physiological model, it is clear that the epithelia responsible for hemolymph ionoregulation are likely to be important for low temperature tolerance of insects^17^,^33^,^34^.

In the present study, we hypothesize that variation in cold tolerance among *Drosophila* species arises from variation in the ability to maintain ion and water balance in the cold, as has been demonstrated in the case of phenotypic plasticity in *D. melanogaster*^18^. Secondly, we hypothesize that maintenance or failure of whole organism ion and water balance is determined, at least in part, by the effects of temperature on MT ion transport rates. We thus predict that if placed at the same low temperature conditions, cold tolerant *Drosophila* species would: 1) better maintain hemolymph volume, 2) better maintain [Na^+^] and [K^+^] balance in their extracellular fluid, and 3) have MT with more stable transport capacities.

## Results and Discussion

**Figure 1:**
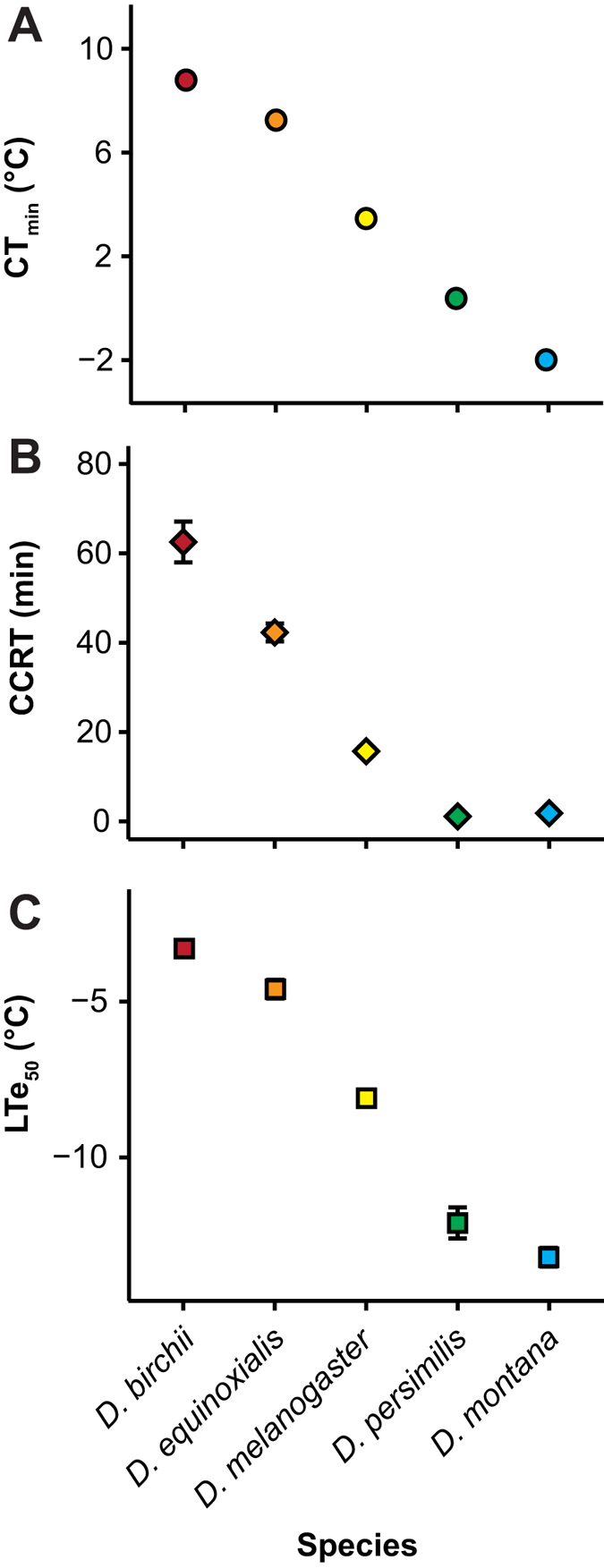
Cold tolerance of the five *Drosophila* species stocks used in this study. Cold tolerance was measured as (**A**) chill coma onset temperature (CT_min_), (**B**) time to stand following 4 h at 0 °C (chill coma recovery time, or CCRT), and (**C**) the temperature that causes 50% mortality (quantified 24 h after a 2 h cold exposure, or LTe_50_). CT_min_ and LTe_50_ values are derived from a recent study on the same fly stocks^6^. Species are colour-coded according to their cold tolerance (warm colours indicate chill susceptible species and cool colours indicate chill tolerant species). All values are mean ± sem. Error bars that are not visible are obscured by the symbols.

Based on previous studies of cold tolerance within the *Drosophila* phylogeny^6^,^7^, five species (*D. birchii*, *D. equinoxialis*, *D. montana, D. melanogaster*, and *D. persimilis*; Table S1) were selected to both minimize phylogenetic bias (all five species come from different subgroups of the genus) and maximize variation in cold tolerance (Fig. 1). All species were reared and maintained as adults for six days under common conditions (20 ± 1 °C, 12:12 L:D), and we quantified cold tolerance as the critical thermal minimum (CT_min_; here meaning the temperature causing complete neuromuscular paralysis, termed chill coma), the temperature that causes 50% mortality from a 2h exposure (LTe_50_), and the time required to stand following a cold exposure (termed chill coma recovery time, or CCRT).

The three measures of chilling tolerance used closely agreed on the variation in chilling tolerance among our five species (R^2^ ≥ 0.96 in all cases; Fig. 1). The rank order of CT_min_ and LTe_50_ values were identical, with *D. birchii* and *D. equinoxialis* being the species most susceptible to chilling, *D. persimilis* and *D. montana* being the most tolerant of cold exposure, and *D. melanogaster* being intermediate in chilling tolerance (Fig. 1)^6^,^35^. The five species differed in their time to recover from 4h at 0 °C (F_4,94_ = 134.7, *P* < 0.001), and CCRT had the same rank order among species as the other measures of cold tolerance, with the exception that *D. montana* and *D. persimilis* both stood almost immediately after (<2 min) being removed from the cold (Fig. 1). To confirm that the flies all entered chill coma during exposure to 0 °C, and determine whether *D. montana* and *D. persimilis* were already capable of standing when removed from 0 °C after 4h, we visually monitored a second set of eight flies of each species during the same cold exposure. All individuals of every species were observed to cease movement entirely (enter chill coma) within 1 min of being placed at 0 °C. After 2h no movement was observed. After 4h at 0 °C, movement was observed in 50% (4/8) of the *D. montana*, and after 6h all of the *D. montana* were standing and 25% (2/8) of the *D. persimilis* were moving (but not standing). None of the other species were observed to resume any movement at 0 °C within 6h. From this observation, we conclude that *D. montana* and *D. persimilis* are physiologically capable of acclimating while in chill coma, and can recover the ability to move and stand (*D. montana*) while still at 0 °C. This ability is likely not limited to these chill tolerant species, as *D. melanogaster* has also been observed to recover from chill coma when held at temperatures between 2 and 4 °C^36^,^37^. We thus suggest that recovering from chill coma during a cold exposure is related to the proximity of the exposure temperature to the species-specific CT_min_. Because the CT_min_, CCRT, and LTe_50_ were all in such close agreement on the relative chill tolerance of the five species, we herein compare all physiological traits to cold tolerance using the LTe_50_ as an index of chilling tolerance. We note, however, that the choice of metric used in these correlations has no impact on our conclusions, and that these traits are often strongly correlated within and among species despite their different physiological underpinnings in relation to ion homeostasis and neuromuscular excitability^6^,^38^.

**Figure 2:**
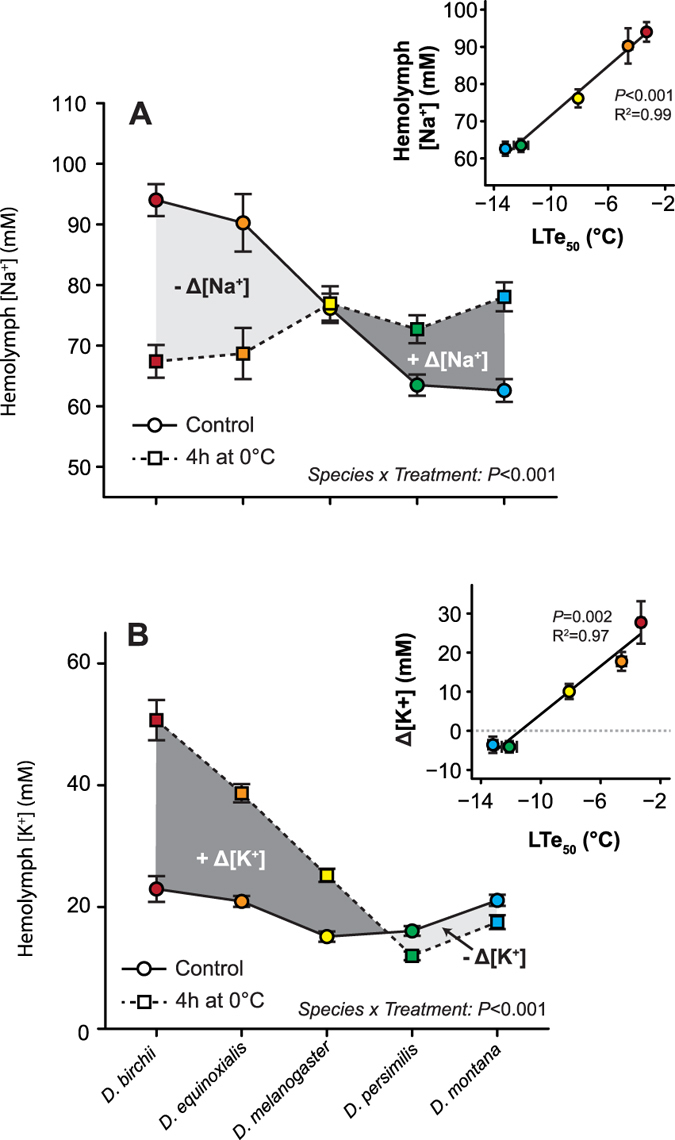
Chill susceptible and chill tolerant *Drosophila* species differ in their ability to maintain ion homeostasis in the cold. Hemolymph Na^+^ (**A**) and K^+^ (**B**) concentrations before (circles with solid black line) and immediately following 4 h at 0 °C (squares with dashed black line) in five *Drosophila* species (crossing lines illustrate significant interaction between species and ion concentration). Solid grey areas illustrate the magnitude effect of cold exposure on concentrations of Na^+^ and K^+^ in the hemolymph. Initial hemolymph [Na^+^] (before any cold exposure was experienced) was a strong predictor of cold tolerance among the five species (shown as LTe_50_; A–insert). Chill susceptible species suffered a loss of ion balance in the cold, as shown by the positive association between cold tolerance and the change in extracellular [K^+^] (Δ[K^+^]) following cold exposure (B–insert). All values are mean ± sem.

It has been reported previously that chill tolerant *Drosophila* species have lower hemolymph Na^+^ and K^+^ concentrations than chill susceptible species under identical rearing conditions^23^. This pattern persists when the phylogeny of the genus is included in the analysis suggesting that low hemolymph [Na^+^] and [K^+^] are adaptive traits and/or are mechanistically associated with thermal tolerance^23^. Within this smaller set of species we here found a similar pattern where hemolymph [Na^+^] varied among the five species (F_4,57_ = 24.5, *P* < 0.001; Fig. 2A) such that hemolymph [Na^+^] (measured prior to cold exposure) had a highly significant negative relationship with cold tolerance (shown as LTe_50_, but relationships were significant regardless of the tolerance measure used; R^2^ = 0.99, df = 3, *P* < 0.001; Fig. 2A insert). The species most tolerant of chilling (*D. montana*) had c. 33% lower hemolymph [Na^+^] under control conditions compared to the least tolerant species (*D. birchii*; Fig. 2A). Initial hemolymph [K^+^] also varied (between c. 15 and 22 mM) among the five species (F_4,55_ = 7.7, *P* < 0.001). For extracellular [K^+^], however, the previously described pattern appears to only hold among four of the five species used in the present study, since *D. montana* (the most cold tolerant species) did not differ from the less tolerant species in their hemolymph [K^+^] (c. 21 mM; Fig. 2B). This suggests that constitutive maintenance of low hemolymph [K^+^], specifically at benign temperatures, is not requisite for chill tolerance in *Drosophila* (linear regression with LTe_50_: R^2^ = 0.20, df = 3, *P* = 0.449).

Cold exposure had dramatic and contrasting effects on extracellular ion balance of the chill tolerant and susceptible *Drosophila* species, respectively. [Na^+^] had decreased considerably in the most susceptible species (*D. birchii* and *D. equinoxialis*) after 4 h at 0 °C, while the more tolerant species (*D. persimilis* and *D. montana*) had elevated extracellular [Na^+^] at the end of the cold exposure (a significant interaction between treatment and species effects on hemolymph [Na^+^]; F_4,111_ = 20.3, *P* < 0.001; Fig. 2A). Accordingly, the net change in [Na^+^] with cold exposure (Δ[Na^+^]) had a very strong negative relationship with cold tolerance (LTe_50_: R^2^ = 0.97, df = 3, *P* = 0.003).

**Figure 3:**
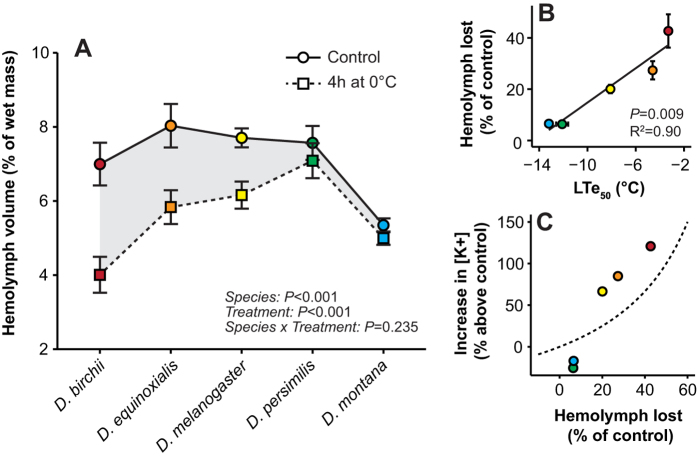
Chill susceptible *Drosophila* species lose hemolymph volume in the cold. (**A**) Hemolymph volume before cold exposure (circles, solid line) and immediately following 4 h at 0 °C (squares, dashed line) in five *Drosophila* species. The shaded grey area denotes the magnitude of hemolymph volume loss during cold exposure (lines illustrate no significant interaction between species and treatment). All values are means ( ± sem). (**B**) The relative disturbance of hemolymph volume correlated positively with cold tolerance (shown as LTe_50_). Error bars that are not clearly visible are obscured by the symbols. All values are means ( ± sem). (**C**) The mean percent increase in hemolymph [K^+^] in relation to the proportion of hemolymph volume lost. Cold exposure caused *D. birchii*, *D. equinoxialis* and *D. melanogaster* to lose K^+^ balance in excess of what is expected from the concentrating effect of hemolymph volume loss alone (dashed line), implying additional K^+^ leak into the hemocoel from the tissues and/or gut lumen. By contrast, *D. montana* and *D. persimilis* reduced hemolymph [K^+^], despite a small reduction in hemolymph volume, suggesting these two species are removing net K^+^ from the hemolymph at 0 °C.

According to our working hypothesis we expected cold exposure to cause a greater leak of Na^+^ ions and water away from the hemolymph in the chill susceptible species and that this would lead to a greater reduction of hemolymph volume in these species. All five species lost hemolymph volume during the cold exposure (F_1,181_ = 21.8, *P* < 0.001, Fig. 3A). Although we found no significant interaction between species and cold treatment on hemolymph volume (Table S2), we observed a trend in the relative volume loss among species; the chill sensitive species suffered a loss of 42% (*D. birchii*) and 27% (*D. equinoxialis*) of their hemolymph water during 4h at 0 °C, while the chill tolerant species (*D. persimilis* and *D. montana*) only lost approximately 6.5% and *D. melanogaster* lost an intermediate proportion of hemolymph water (20%). As was the case for hemolymph [Na^+^], the degree of hemolymph volume disruption observed among the five species was closely related to their observed cold tolerance (R^2^ = 0.93, df = 3, *P* = 0.009; Fig. 3B).

The working hypothesis presented here also dictates that [K^+^] will increase during cold in response to the loss of hemolymph volume loss (which concentrates the remaining K^+^) and/or if passive leakage of K^+^ towards the hemolymph increases above the active removal rate. This was confirmed by the significant interactive effects of treatment and species on hemolymph [K^+^] (F_4,111_ = 39.8, P < 0.001) demonstrating that cold exposure induced varying degrees of [K^+^] change among the species. Exposure to low temperature increased hemolymph [K^+^] in *D. birchii*, *D. equinoxialis,* and *D. melanogaster* (up to 50 ± 3, 39 ± 1, and 25 ± 2 mM, respectively) while *D. persimilis* and *D. montana* actually had lower hemolymph [K^+^] after 4 h at 0 °C than before the cold exposure. The net change in hemolymph [K^+^] (Δ[K^+^]) thus had a strong positive relationship with the LTe_50_ (R^2^ = 0.97, df = 3, *P* = 0.002; Fig. 2B, insert) and was also closely related to the species specific volume loss (R^2^ = 0.98, df = 3, *P* < 0.001; not shown).

To our knowledge, the present study is the first to examine whether differences in the ability to maintain hemolymph water and ion balance at low temperature are likely to underlie interspecific variance in *Drosophila* cold tolerance. These interspecific patterns strongly agree with earlier evidence where cold stress is consistently observed to disrupt water balance and increase hemolymph [K^+^] in a variety of distantly related insect species that, in addition to *Drosophila,* include cockroaches, locusts, crickets, and fire bugs^16^,^17^,^18^,^20^,^21^. Likewise, improvements in insect cold tolerance induced by acclimation are repeatedly associated with an improved ability to maintain low extracellular [K^+^] at low temperatures^18^,^20^,^23^,^39^. The physiological differences that allow chill tolerant species to maintain ion and water balance and cause susceptible species to fail in the cold must therefore be associated with evolved differences in the ability to balance passive and active transport of ions and water at low temperatures. One important factor in this seems to be the initial [Na^+^] concentration as this permeable ion represents an important osmolyte that will tend to leave the hemolymph at low temperature when active transport and reabsorption is compromised. Species with high [Na^+^] therefore lose [Na^+^] to the gut or other tissues and when water follows osmotically, [K^+^] will increase in the progressively decreasing hemolymph volume^16^,^17^,^18^. If the decrease in hemolymph volume observed drives the entirety of the [K^+^] increase then we should expect, for example, that a 50% reduction in volume would cause a 100% increase in ion concentration (Fig. 3C). This is seemingly not the only factor causing hyperkalemia in *Drosophila*, as the observed volume reduction only explained 40–60% of the increases in hemolymph [K^+^] found in the three chilling susceptible species (*D. birchii*, *D. equinoxialis* and *D. melanogaster*, which all increased hemolymph [K^+^] during the cold exposure). Accordingly, water regulation plays an important role for ion homeostasis but some of the increase in [K^+^] must also be caused by leaking from the tissues or gut contents towards the hemolymph, either as a result of tissue injury^32^ or a net leak of K^+^ ions down their concentration gradient.

Muscle function and excitability is highly dependent on the K^+^ equilibrium potential (E_K_) in many insects since the transmembrane distribution of K^+^ largely determines the membrane potential^19^,^28^,^30^. Low temperature and increasing hemolymph [K^+^] both depolarize insect muscles and this depolarization is thought to cause both chill coma onset and chilling injury^19^,^35^,^40^,^41^. In contrast to the chill sensitive species we found that *D. persimilis* and *D. montana* reduced hemolymph [K^+^] during the cold exposure, with little associated change in hemolymph volume, and this is consistent with the observation that these species partially recovered from coma while maintained at a low temperature.

**Figure 4:**
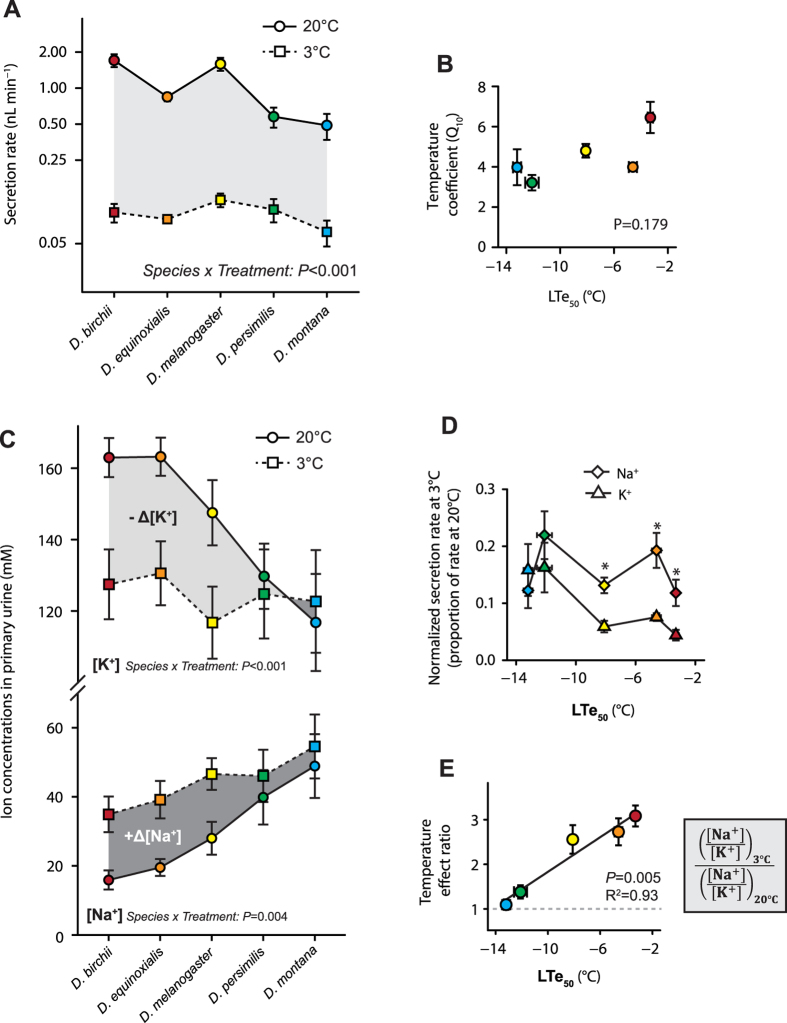
Low temperature strongly suppresses Malpighian tubule (MT) secretion rates in all five *Drosophila* species but only disrupts ion selectivity of the Malpighian tubules of chill sensitive *Drosophila*. (**A**) Rates of primary urine production at 20 °C (circles, solid line) and 3 °C (squares, dashed line) in five *Drosophila* species. Temperature strongly impacted tubule secretion rates in all five species. (**B**) The five species differed in the thermal sensitivity (temperature coefficient; Q_10_) of tubule secretion rates, but there was no clear relationship between thermal sensitivity of urine production and thermal tolerance (thermal tolerance shown here as LTe_50_: the 2 h exposure temperature that causes 50% mortality within 24 h). NB: Secretion rates are plotted on a logarithmic scale. (**C**) Concentrations of Na^+^ and K^+^ in the primary urine produced by the Malpighian tubules of five *Drosophila* species at 20 °C (circles, solid line) and 3 °C (squares, dashed line). In chill susceptible species (*D. birchii, D. equinoxialis, and D. melanogaster*) low temperature decreased the concentration of K^+^ in the primary urine and increased the concentration of Na^+^ while selectivity was only marginally impacted in the chill tolerant species. Temperature and species interacted to affect the [Na^+^] and [K^+^] of the secreted fluid (see Table S2). (**D**) Normalized ion secretion rates at 3 °C (a proportion of ion secretion rates at 20 °C) showing that exposure to low temperature affected rates of Na^+^ and K^+^ secretion differently among the species. The effects of temperature on Na^+^ (diamonds) and K^+^ (triangles) secretion rates for each replicate tubule were calculated from rates of fluid production and ion concentrations in the fluid secreted at 20 °C and 3 °C (see Fig. S1). Asterisks indicate species for which K^+^ secretion was more strongly suppressed at low temperature than Na^+^ secretion based on paired t-tests; *P* < 0.05). (**E**) A calculated “temperature effect ratio” (y-axis; an index of the magnitude of temperature effects on the ratio of ions secreted) regressed against the lower lethal temperatures of the five species. All values shown are mean ± sem and error bars that are not clearly visible are obscured by the symbols.

Clearly, chill tolerant *Drosophila* species have a greater ability to maintain whole organism ion and water homeostasis at low temperatures. Low temperature strongly suppressed rates of primary urine production, in all five species (Fig. 4A,B). Critically, however, the MTs of chill tolerant *Drosophila* differ from those of the chill sensitive species in their ability to secrete constant concentrations of ions in the primary urine at high and low temperatures (Fig. 4C). At benign temperatures the MT typically secrete primary urine with a high [K^+^] and low [Na^+^] (e.g.^42^; Fig. 4C) but at low temperature (3 °C), we observed that the chill susceptible species secrete a higher concentration of [Na^+^] and less [K^+^] in the cold, while chill tolerant species secrete a similar ratio of these ions at 20 and 3 °C (species by temperature interactions; Na^+^: *F*_4,49_ = 4.4, *P* = 0.004, K^+^: *F*_4,49_ = 7.5, *P* < 0.001; Fig. 4C).

By combining the rates of primary urine production from the tubules and the ion concentrations in the secreted fluid, we calculated Na^+^ and K^+^ secretion rates (pmol min^−1^) at 20 °C and 3 °C. Temperature interacted with species to influence total rates of both Na^+^(interaction: *F*_4,49_ = 5.4, *P* = 0.001) and K^+^ secretion (interaction: *F*_4,49_ = 13.5, *P* < 0.001), but suppressed Na^+^ and K^+^ secretion in all five species (Fig. S1). Notably, however, the three more chill sensitive species (*D. birchii*, *D. equinoxialis* and *D. melanogaster*), all suffered a greater reduction in K^+^ secretion (approximately 2–3% of rates at 20 °C) than Na^+^ secretion (approximately 5–10% of rates at 20 °C; Fig. 4D). To quantify the overall effect of temperature on the ratio of ions secreted in the primary urine, we calculated the [Na^+^]:[K^+^] at 3 °C relative to 20 °C. The MT’s of *D. birchii* and *D. equioxialis* secrete roughly three times as much Na^+^(relative to K^+^) at 3 °C as at 20 °C, while the ratios of ions secreted by *D. persimilis* and *D. montana* tubules are far less affected by low temperature, such that the overall effect of temperature on the ratio of ions secreted was closely related to the LTe_50_ (linear regression: R^2^ = 0.93, *d.f.* = 3, *P* = 0.005; Fig. 4E).

Given that the more cold tolerant species maintained constitutively lower hemolymph [Na^+^], we questioned whether the observed differences in temperature effects on ion transport among species was artificially produced by the use of a constant and intermediate [Na^+^] in the bathing saline. A supporting experiment, however, confirmed that reducing the [Na^+^] of the bathing saline had no impact on the rates of primary urine production, nor on the temperature effects on secretion rates in either *D. birchii* (chill susceptible) or *D.persimilis* (chill tolerant; Fig. S2). Halving [Na^+^] in the bathing saline did, however, reduce the average [Na^+^] in the secreted fluid by c. 50% in both species (Although this effect was only statistically significant in *D. persimilis* (*D. birchii*: *F*_1,18_ = 2.8, *P* = 0.111; *D. persimilis*: *F*_1,18_ = 5.3, *P* = 0.034; Fig. S2)).

**Figure 5:**
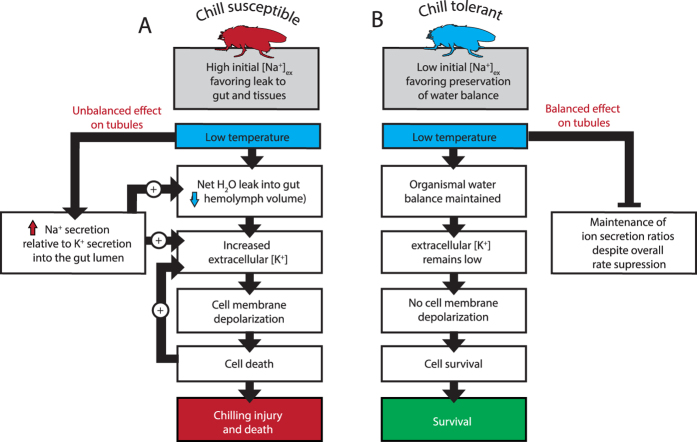
A conceptual model of how chilling differentially affects chill-susceptible and chill tolerant insects. (**A**) Chill susceptible insects maintain large [Na^+^] gradients, such that Na^+^ is a dominant extracellular osmolyte. At low temperatures, net Na^+^ leak across the gut epithelia causes a disruption of water balance, as extracellular water moves to the gut lumen. This disruption is partly driven by the effects of low temperature on the Malpighian tubules, which secrete a greater proportion of Na^+^ into the gut lumen relative to K^+^ in the cold (Fig. 4). Ultimately, the loss of hemolymph volume and impaired ability to clear K^+^ concentrate K^+^ in the extracellular space, causing cell depolarization and cell death (which likely causes further K^+^ leak into the extracellular space from compromised cells in a positive-feedback loop^32^). By contrast, chill tolerant insects (**B**) maintain lower Na^+^ gradients (making water balance less dependent on Na^+^) and defend the ratio of ions secreted by the Malpighian tubules in the cold, despite overall slowing of secretion rates at low temperatures. As a consequence of these, and likely several other adaptations, the tolerant species maintain organismal water and ion balance at low temperatures (or even bolster existing gradients; Fig. 2) and recover from the same cold stress with little to no observable injury.

Overall, the observed effects of temperature on renal transport serve to explain, at least in part, the differential effects of low temperature on organismal ion and water balance (Figs 2 and 3). Specifically, secretion of a higher concentration of Na^+^ relative to K^+^ in the cold will reduce hemolymph Na^+^ content. Because Na^+^ is one of the principal osmolytes in these species the removal of Na^+^ causes a net loss of water from the hemolymph, which contributes to the observed increase in extracellular [K^+^] (Fig. 5). This tendency for increased extracellular [K^+^] during cold exposure is exacerbated by the relatively stronger reduction in K^+^ clearance by the Malpighian tubules of the chill susceptible species (Fig. 5). By contrast, the chill tolerant species (*D. persimilis* and *D. montana*) constitutively maintain low hemolymph [Na^+^], which contributes to cold tolerance by limiting rates of [Na^+^] leak at low temperatures. Furthermore, these species possess MT’s that are similarly slowed by cooling, but importantly maintain their typical selectivity for K^+^ and Na^+^. This enables the chill tolerant species to avoid water balance disruption, maintain hemolymph ion balance, and quickly recover from the same cold stress without any observable injury (Fig. 5).

If our *in vitro* results are translated to *in vivo* capacity, the *Drosophila* MTs are capable of clearing all of the animal’s hemolymph water or potassium ions within minutes. Cold reduces this transport capacity significantly for all species, but temperature has a balanced effect on Na^+^ and K^+^ transport in the cold tolerant species, and more strongly suppresses K^+^ transport in the chill sensitive species. These observed differences in the temperature sensitivity of MT selectivity appear adaptive but it is also clear that ion balance *in vivo* is influenced by other factors, such as ion and water reabsorption rates in the gut epithelia and changes in ion permeability of cells and epithelia. Accordingly, we suggest that the ability of the MTs to secrete a constant ratio of ions at benign and low temperatures is only one of several adaptations associated with increased chill tolerance. Chill tolerant species may also, for example have a greater capacity for ion regulation in the hindgut (the other major ionoregulatory organ). Thermal adaptation of secretion and absorption processes will likely be related to changes to the quaternary structure of ion transport proteins^23^, the regulation of such proteins through signaling pathways^33^, modulation of the membrane environment in which those transporters sit^43^,^44^, and/or modifications to transcellular and paracellular ion and water permeability^45^. As Na^+^/K^+^-ATPase activity in the MT basolateral membrane of *D. melanogaster* larvae has been demonstrated to impact the relative concentrations of Na^+^ and K^+^ in the secreted fluid^46^, failure of this ubiquitous ion transporter may be of particular importance in determining the observed temperature effects on MT transport. Curiously, however, a larger analysis of 24 *Drosophila* species found no relationship between the thermal sensitivity of Na^+^/K^+^-ATPase activity in whole-animal homogenates and organismal thermal tolerance, and cold-acclimated *Drosophila* constitutively maintain less, rather than more, Na^+^/K^+^-ATPase protein in an active state^23^.

In conclusion, we have outlined a comprehensive working model of how chill tolerance is intimately linked to homeostatic regulation of ion and water-balance, in the most common model insect, *Drosophila* (Fig. 5). A failure of cold-sensitive species to maintain, ion and water balance causes a dramatic increase in extracellular [K^+^], which compromises neuromuscular function and causes injury. Further we have shown how the ability of cold tolerant species to maintain homeostasis is facilitated (in part) by their renal systems, which continue to secrete ions in a constant ratio (albeit at a slower rate) in the cold. In fact, we observed that chill tolerant species were even able to decrease their hemolymph [K^+^] during cold exposure and we speculate that this can explain the progressive recovery of muscular function that we observed at 0 °C in these species. We emphasize that the differences observed here represent innate differences in the function of the tubules at low temperature among these five species; our tubule assays were completed in the absence of any stimulation from insect neuropeptides that can influence primary urine production^33^,^47^. Indeed, Capa (a neuropeptide which targets the Malpighian tubules) has already been demonstrated to impact rates of chill coma recovery in *D. melanogaster*, possibly through modulation of tubule transport rates upon rewarming^33^. Ultimately, the foundational knowledge presented here allows for detailed study into how renal functions are impacted by, and regulated at, low temperature, and how other aspects of water and ion-balance differ among species that vary in cold tolerance. We expect such an approach will uncover novel molecular mechanisms of thermal adaptation and acclimation.

## Materials and Methods

### Maintenance and rearing of experimental animals

The five species of *Drosophila* used in this study were derived from private and commercial laboratory stocks (Table S1) that are maintained under “common garden” conditions at 20 ± 1 °C, and a 12:12 h light:dark cycle. Between 50 and 250 parental flies were kept in 250 ml bottles containing 50 ml of oat-based Leeds medium (60 g yeast, 40 g sucrose, 30 g oatmeal, 16 g agar, 12 ml methylparaben (a fungicide) and 1.2 ml acetic acid per liter of water). Mature adults were transferred to fresh bottles with a small amount of dry yeast, and allowed to oviposit for between 2 and 48h (depending on species) before being removed, which allowed us to carefully control larval rearing density of experimental flies (100–150 per bottle). Flies that emerged from these bottles were removed daily, and transferred to 35 ml vials containing 4 ml of the same medium and a piece of filter paper (25–50 flies per vial). Flies were left to mature for three days in vials before being sorted by sex under light CO_2_ anesthesia. Female flies were transferred to fresh vials and left for a further 3–4 days to recover from the CO_2_ exposure and further mature before being used for experiments^48^,^49^, so all of the female flies used were assumed to be non-virgins, and were between six and seven days of age when used for measurements.

### Cold tolerance

Cold tolerance was measured as chill coma recovery time (CCRT): the time required to recover the ability to stand following 4 h at 0 °C as previously described^6^. Briefly, twenty flies of each species were individually transferred to 5 ml glass vials that were sealed and immersed directly into a mixture of ice and distilled water (0 °C), where they remained for 4 h. The vials were then quickly removed from the cold, and arranged on a laboratory bench at 23 °C. Flies were continuously observed, without being physically disturbed, until they spontaneously righted themselves. The time from removal of the vial from the cold to spontaneous righting was quantified as CCRT. We noted that *D. persimilis* and *D. montana* (the most chill tolerant species) recovered very rapidly from chill coma (main document - Fig. 1). To confirm that these species entered chill coma at 0 °C, and whether they recovered from chill coma during the cold exposure, n = 8 flies of each species were transferred to 5 mL glass vials and submerged in an ice-water slurry in an glass aquarium that was constantly mixed by a small pump and held at 0 °C by regular additions of ice. The flies were monitored upon first being placed into the aquarium, and checked after 2, 4 and 6 h at 0 °C, by taping on the flies and observing them with a magnifying glass for any signs of movement or posture.

The critical thermal minima (CT_min_) of the five *Drosophila* species was measured by gradually cooling the flies while simultaneously observing their ability to move, and have been published as part of a recent and related study on the same five species stocks^35^. Briefly, twenty flies of each species were individually placed in 5 ml sealed containers, mounted on a rack, and submerged in a bath set to 20 °C and filled with a transparent, ethylene-glycol and water mixture (1:2). The temperature was then gradually decreased (0.2 °C min^−1^) and the flies were continuously observed. When the flies ceased spontaneous movement they were stimulated to move by tapping on the vial, and the chill coma temperature (here termed CT_min_) was registered when no response could be observed.

Data on lower lethal temperatures of the five species were taken from a previous study on the same stocks^6^, and collected approximately one year prior to the data in the present study. In that study, temperatures that caused 50% mortality (LTe_50_–2 h) were determined from survival curves of flies exposed to 6–12 different low temperatures for 2 h, and survival was assessed 2 h after removal from the cold to room temperature (23 °C).

### Hemolymph ion concentrations

Ion concentrations were measured in the hemolymph (n = 11–14 per species and treatment group for each ion) as previously described^18^. Hemolymph was sampled from adult flies that were taken either directly from their rearing conditions (20 °C), or following 4 h at 0 °C. The cold exposure was conducted as in the chill coma recovery experiment, except that flies were pre-loaded into 10-μL pipette tips (sealed with parafilm) to facilitate rapid hemolymph collection by antennal ablation as described below.

To collect hemolymph, flies were positioned headfirst in a 10-μL pipette tip attached to a tubing system which applied positive air pressure from behind *via* a laboratory air supply. The end of the pipette tip was cut to expose the antennae and one of the antennae was then ablated at its first segment, whereupon a clear droplet of hemolymph flowed out of the wound due to the positive air pressure applied on the body of the fly^50^. The pipette tip (still containing the fly and attached droplet) was then removed from the device, and the droplet was isolated under hydrated paraffin oil to allow for immediate measurement of Na^+^ or K^+^.

Physiologically active concentrations (activities) of Na^+^ and K^+^ were measured using ion-selective microelectrodes in hemolymph droplets from mutually exclusive sets of flies. Ion selective electrodes were constructed from borosilicate glass capillaries (TW-150-4, World Precision Instruments (WPI), Sarasota, FL, USA) that were pulled to a tip diameter of ~3 μm with a P-97 Flaming Brown micropipette puller (Sutter Instruments Co., Novato, USA). Glass micropipettes were heated to 300 °C and exposed to N,N-dimethyltrimethylsilylamine vapour for for 1 h. Potassium sensitive electrodes were made by backfilling the treated glass with 100 mM of KCl and front-filling with K^+^ ionophore (K^+^ ionophore I, cocktail B, Sigma Aldrich, St. Louis, MO, USA). Sodium sensitive electrodes were made by backfilling with 100 mM NaCl and front-filling with a with an Na^+^ ionophore cocktail [Na^+^ ionophore X;^51^]. Electrodes were then dipped in a solution of polyvinylchloride mixed in tetrahydrofuran, to prevent displacement of the ionophore^52^. An FD223a differential electrometer (WPI) was used to record the output voltage which was digitalized using a MP100A data acquisition system and collected using AcqKnowledge software (Biopac Systems, Goleta, CA, USA). The circuit was completed using a glass reference electrode (IB200F-4, WPI) backfilled with 0.5 M KCl. Voltage measurements in hemolymph droplets were converted to ion activity through reference to calibrations solutions with a ten-fold concentration difference in the target ion (Na^+^: 15 and 150 mM; K^+^: 10 and 100 mM; the concentration difference between the two solutions was made up with LiCl in both cases). Voltages from the ion-selective electrode were converted to ion concentrations using equation (1):


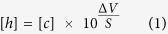


where [*h*] is the active ion concentration in the hemolymph, [*c*] is the concentration in one of the calibration solutions, Δ*V* is the voltage difference between the calibration solution and hemolymph, and *S* is the slope of the voltage response to the ten-fold concentration difference in the calibration solutions. The slopes of Na^+^- and K^+^-selective electrodes used were 56.2 ± 3.0 and 54.4 ± 1.6 mV per 10-fold difference in ion concentration, respectively (mean ± s.d.), close to the expected Nernst relationship of 58 mV.

### Hemolymph volume

We measured hemolymph volume in *Drosophila* using a previously described gravimetric blotting method in n = 18–21 flies per species and experimental group. Briefly, control flies (those that did not receive a cold exposure) were anaesthetized under light CO_2_ for 10 s before being rapidly weighed to determine wet mass (WM). Flies that received a cold exposure (in chill coma) were taken directly from the cold and weighed without CO_2 _anaesthesia. Individual flies (regardless of treatment) were then placed on the CO_2_-releasing pad in the field of view of a dissecting microscope. Small triangular pieces of filter paper (ca. 0.5 cm^2^) were dried at 60 °C in an oven and the corner of the paper was pre-wet with 300 mM sucrose solution. Fine forceps were used to open the ventral abdomen of the fly, and the corner of the paper was inserted into the opening in the fly. Hemolymph was observed to soak into the paper by capillary action for 15 s, whereupon the paper was removed and the fly was reweighed to determine hemolymph-free wet mass (HFWM). Hemolymph mass was calculated as the difference between WM and HFWM.

### Malpighian tubule secretion rates

Secretion rates of the Malpighian tubules were determined *in vitro* at 20 and 3 °C using the Ramsay assay^53^,^54^. Flies of each species were placed on a mixture of ice and water (0 °C) for 10 s to induce a brief chill coma (simply to incapacitate the flies) before they were submerged in a 1:1 mixture of *Drosophila* saline (132 mM Na^+^, 20 mM K^+^, 158 mM Cl^−^, 8.5 mM Mg^2+^, 2 mM Ca^2+^, 10.2 mM HCO_3_^−^, 4.3 mM H_2_PO_4_^−^, 22 mM glucose, 8,6 mM HEPES, pH 6.8) and Schneider’s *Drosophila* medium^54^ (final Na^+^ and K^+^ conditions: 89 mM Na^+^, 22 mM K^+^). The head was quickly removed and the Malpighian tubules were dissected away from the remainder of the gut at 23 °C, before they were transferred to a dish containing fresh buffer. The preparation dish was filled with paraffin oil and suspended in a double-walled glass dish. A 1:1 mixture of ethylene glycol was circulated through the glass dish by a refrigerated circulator, which allowed for precise temperature control of the oil surrounding the tubule preparations. The anterior tubules, including the lower tubule^42^, were suspended in a 10 μl droplet of the above-mentioned saline, containing 40 μM amaranth dye, which is an innocuous red food dye that is regularly used to help visualize droplets in Ramsay assays^54^. The posterior tubules were wrapped around a nearby pin (outside the droplet), such that the ureter (with a minimal remaining portion of the gut) was free from the saline. In a subsequent experiment on *D. birchii* and *D. persimilis*, tubules were prepared as described above, but the Na^+^ concentration in the final buffer was reduced from 89 mM to 50 mM, with the resulting difference in saline osmolality corrected by additional glucose.

Early trials confirmed that tubule secretion rates were highly stable over time [as has been previously reported for *D. melanogaster*^54^] and that the order of temperatures used did not impact secretion rates. Thus, we chose to expose all tubules to decreasing temperatures (20 °C first, followed by 3 °C). Tubules were given 15 min to rest at 20 °C before the experiment began. Droplets were then removed approximately every 15 minutes (n = 2 droplets per tubule), before the temperature was reduced to 3 °C for approximately 1.5 h before another droplet was removed for the 3 °C measurement. Droplet size was determined by moving each droplet to a common “staging area” next to a pin of known diameter. Each droplet was photographed (along with the pin) at 50× magnification. Droplet diameter was quantified in Image J and used to calculate volume and (with time) rates of primary urine production. Temperature coefficients (Q_10_) of secretion rates were calculated from the 20 °C and 3 °C secretion rates of each tubule.

### Data analysis

All data analysis was done in the R environment for statistical computing (version 3.1)^55^. Differences in chill coma recovery time among species were analyzed using analysis of variance (ANOVA) followed by Tukey’s HSD. Ion concentrations and hemolymph volume (in μL) were analyzed using linear models, with species and treatment (control or cold exposed), and body mass (only in the case of hemolymph volume) included as factors and covariates where appropriate. Log_10_-transformed Malpighian tubule fluid secretion rates (nL min^−1^), ion secretion rates (pmol min^−1^) and ion concentrations in the secreted fluid were all analyzed using linear mixed effect models by the lmer() function in the lme4 package for R. Species and temperature were treated as fixed factors and the individual tubule was included as a random factor. Relationships between physiological traits (e.g. mean changes in ion concentration (Δ[ion]), hemolymph volume change (proportion of body mass), thermal sensitivity of tubule secretion rates, or “temperature effect ratio” of the ions secreted in the primary urine) and thermal tolerance (LTe_50_) were analyzed by linear regression.

## Additional Information

**How to cite this article**: MacMillan, H. A. *et al.* The capacity to maintain ion and water homeostasis underlies interspecific variation in *Drosophila* cold tolerance. *Sci. Rep.* **5**, 18607; doi: 10.1038/srep18607 (2015).

## Supplementary Material

Supplementary Information
