# Supplementary material for: The capacity to maintain ion and water homeostasis underlies interspecific variation in Drosophila cold tolerance
Source: Sci Rep. 2015 Dec 18;5:18607. doi: 10.1038/srep18607 (PMC4683515; doi:10.1038/srep18607)
Supplement: Supplementary Information [file srep18607-s1.pdf]

SUPPLEMENTARY MATERIAL

The capacity to maintain ion and water homeostasis underlies interspecific variation in *Drosophila* cold tolerance

Heath A. MacMillan[1*], Jonas L. Andersen[1], Shireen A. Davies[2], and Johannes Overgaard[1]

1. Zoophysiology, Department of Bioscience, Aarhus University, Aarhus, Denmark
2. Institute of Molecular, Cell and Systems Biology, College of Medical, Veterinary and Life Sciences, University of Glasgow, Glasgow, United Kingdom.

*Author for correspondence: macmilla@yorku.ca

Present address: Department of Biology, York University, Toronto, Canada

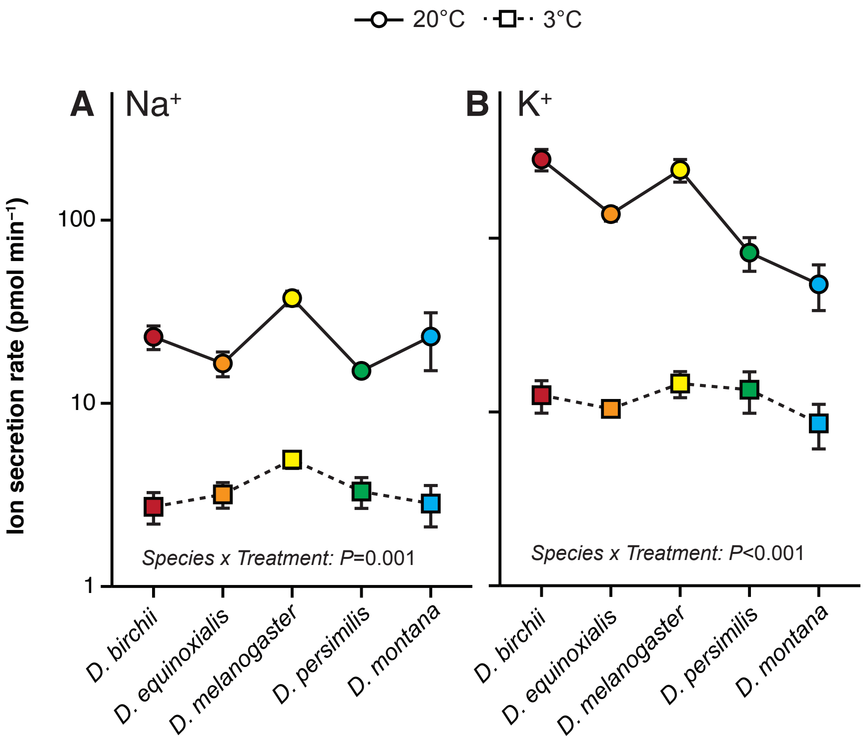

Fig S1. Interactive effects of species and temperature impact total rates of ion secretion by the Malpighian tubules in *Drosophila*.

Secretion rates of Na^+ (A) and K^+ (B) were calculated from rates of fluid secretion and ion concentrations in the fluid secreted by adult females of five species of *Drosophila*. Reducing temperature from 20°C (circles, solid black line) to 3°C (squares, dashed black line) caused a substantial decrease in rates of secretion of both ions, but the magnitude of this effect depended on the species. All values are mean ± sem.

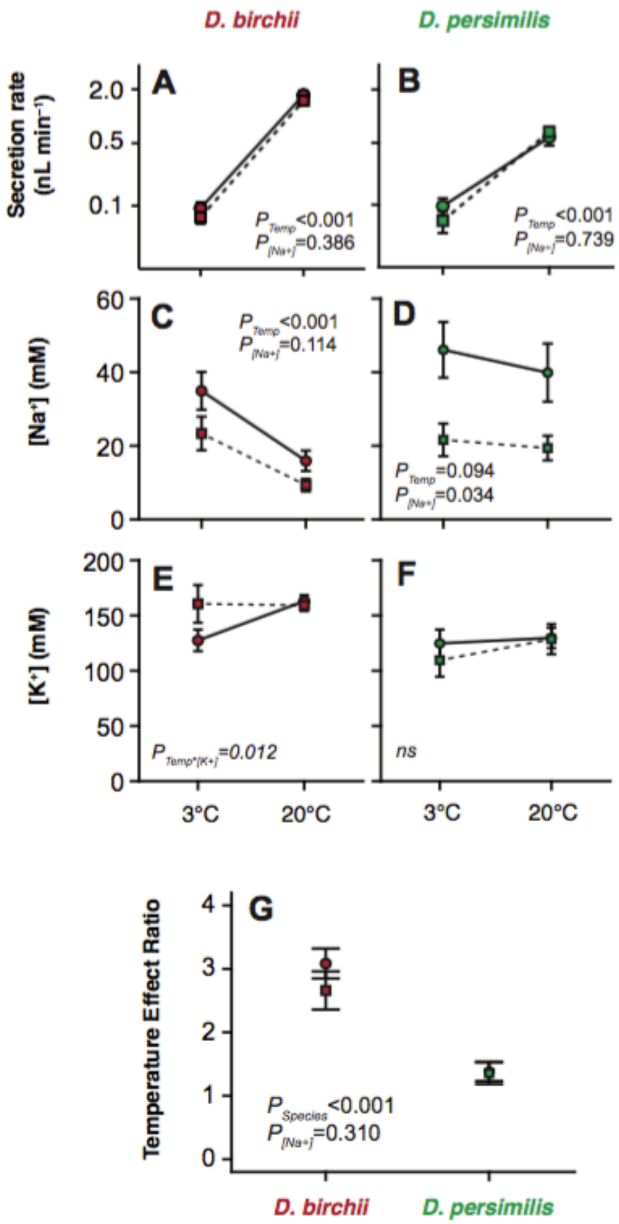

Fig S2. The bathing saline [Na^+] impacts rates of Na^+ secretion, but not the thermal sensitivity of Malpighian tubule water or ion secretion.

Rates of primary urine production (A,B), concentrations of Na^+ (C,D) and K^+ (E,F), and the effect of temperature on the ratio of ions secreted (G) from tubules of *D. birchii* (red, chill susceptible) and *D. persimilis* (green; chill tolerant). Tubules were bathed in a saline containing either 77 mM Na^+ (circles; similar to *D. melanogaster* hemolymph, and as used in other tubule assays) or 38 mM Na^+ (squares), with the resulting difference in osmolality corrected by additional glucose. Reducing [Na^+] in the bathing saline reduced the [Na^+] or the secreted fluid in both species by c. 50%, regardless of temperature, but had no significant impact on the effect of temperature on primary urine production rates nor the ratio of ions secreted. Error bars that are not clearly visible are obscured by the symbols.

Table S1. Details of the five *Drosophila* species used. All species were reared and maintained at 20±1°C for several generations prior to experiments. The *Drosophila* species stocks were originally provided from laboratory cultures by: Professor Anneli Hoikkala, University of Jyväskylä, Finland (*D. montana*); Professor Volker Loeschcke, Aarhus University, Denmark (*D. melanogaster*); the *Drosophila* Species Stock Center, San Diego, USA (*D. equinoxialis and D. persimilis*) and Professor Ary Hoffmann, University of Melbourne, Australia (*D. birchii*).

Species (abbreviation)	Origin	Collection date	Distribution	Source
***D. birchii* (bir)**	Australia	2008	Tropical	Hoffmann, AU
***D. equinoxialis* (equ)**	Honduras	<1984	Tropical	DSSC, US
***D. melanogaster* (mel)**	Denmark	2011	Cosmopolitan	Loeschcke, DK
***D. persimilis* (per)**	Canada	Unknown	Temperate	DSSC, US
***D. montana* (mon)**	Finland	2008	Temperate	Hoikkala, FI

Table S2. Results of generalized linear models (GLM) on the independent and interactive effects of species and cold exposure treatment on hemolymph volume and ion concentrations, and results of linear mixed effects models (LMEM) on the independent and interactive effects of temperature and species in Malpighian tubule secretion rates ion concentrations of the secreted fluid, and absolute ion secretion rates. Factors or interactions shown in bold typeface had a significant effect on the dependent variable.

Variable	Test	Covariates/ Random effects	Factors	F	df	*P*-value	Sig.
**Hemolymph Na+**	GLM	None	**Species**	**8.0**	**4,111**	**<0.001**	***
			**Treatment (Control/4h at 0°C)**	**6.2**	**1,111**	**0.014**	*
			**Species × Treatment**	**20.3**	**4,111**	**<0.001**	***
**Hemolymph K+**	GLM	None	**Species**	**68.5**	**4,111**	**<0.001**	***
			**Treatment (Control/4h at 0°C)**	**100.4**	**1,111**	**<0.001**	***
			**Species × Treatment**	**39.8**	**4,111**	**<0.001**	***
**Hemolymph volume**	GLM	Body mass	**Species**	**16.2**	**4,181**	**<0.001**	***
			**Treatment (Control/4h at 0°C)**	**21.8**	**1,181**	**<0.001**	***
			Species × Treatment	1.4	4,181	0.235	ns
**Rate of urine production (nL min-1)**	LMEM	Tubule	Species	0.5	4,95	0.756	ns
			**Temperature**	**204.3**	**1,49**	**<0.001**	***
			**Species × Temperature**	**14.4**	**4,49**	**<0.001**	***
**Urine [K+] (mM)**	LMEM	Tubule	Species	1.5	4,49	0.224	ns
			**Temperature**	**43.4**	**1,49**	**<0.001**	***
			**Species × Temperature**	**7.5**	**4,49**	**<0.001**	***
**Urine [Na+] (mM)**	LMEM	Tubule	**Species**	**3.0**	**4,49**	**0.026**	*
			**Temperature**	**81.5**	**1,49**	**<0.001**	***
			**Species × Temperature**	**4.4**	**4,49**	**0.004**	**
**Na+ secretion rate (pmol min-1)**	LMEM	Tubule	**Species**	**6.2**	**4,49**	**0.004**	**
			**Temperature**	**144.2**	**1,49**	**<0.001**	***
			**Species × Temperature**	**5.4**	**4,49**	**0.001**	**
**K+ secretion rate (pmol min-1)**	LMEM	Tubule	**Species**	**11.0**	**4,49**	**<0.001**	***
			**Temperature**	**150.6**	**1,49**	**<0.001**	***
			**Species × Temperature**	**13.5**	**4,49**	**<0.001**	***

Table S3. Results of linear mixed effects models on the independent and interactive effects of temperature and buffer sodium concentration on Malpighian tubule secretion rates and ion concentrations of the secreted fluid. Fixed factors in bold typeface had a significant effect on the dependent variable.

Species	Dependent variable	Test	Random effects	Fixed factors	*F*	df	*P*-value	Sig.
D.birchii	Secretion rate (nL min^{-1})	LMEM	Tubule	Buffer Na+	0.786	1,18	0.386	ns
				**Temperature**	**116.829**	**1,18**	**<0.001**	***
				Buffer Na+ × Temperature	0.625	1,18	0.432	ns
	Urine [K+] (mM)	LMEM	Tubule	Buffer Na+	1.3962	1,18	0.252	ns
				**Temperature**	**6.7031**	**1,18**	**0.018**	*
				**Buffer Na+ × Temperature**	**7.7581**	**1,18**	**0.012**	*
	Urine [Na+] (mM)	LMEM	Tubule	Buffer Na+	2.81	1,18	0.111	ns
				**Temperature**	**61.362**	**1,18**	**<0.001**	***
				Buffer Na+ × Temperature	1.384	1,18	0.255	ns
D.persimilis	Secretion rate (nL min^{-1})	LMEM	Tubule	Buffer Na+	0.114	1,18	0.74	ns
				**Temperature**	**67.352**	**1,18**	**<0.001**	***
				Buffer Na+ × Temperature	0.831	1,18	0.374	ns
	Urine [K+] (mM)	LMEM	Tubule	Buffer Na+	0.2388	1,18	0.631	ns
				Temperature	4.0558	1,18	0.059	ns
				Buffer Na+ × Temperature	1.401	1,18	0.252	ns
	Urine [Na+] (mM)	LMEM	Tubule	**Buffer Na+**	**5.2806**	**1,18**	**0.034**	*
				Temperature	3.1162	1,18	0.094	ns
				Buffer Na+ × Temperature	0.6915	1,18	0.416	ns
